# Macular Vascular Density Changes following Cataract Surgery in Diabetic Patients: An Optical Coherence Tomography Angiography Study

**DOI:** 10.1155/2021/6641944

**Published:** 2021-03-26

**Authors:** Le Feng, Guliqiwaer Azhati, Tingting Li, Fang Liu

**Affiliations:** Department of Ophthalmology, Shanghai Tenth People's Hospital, Tongji University School of Medicine, Shanghai, China

## Abstract

**Purpose:**

Cataracts and diabetes very commonly coexist. The aim of the present study was to quantify the effect of uncomplicated phacoemulsification on retinal microvasculature in diabetic patients by using optical coherence tomography angiography (OCTA).

**Methods:**

A prospective observational study of diabetic and nondiabetic patients at baseline and 1 day, 1 week, 1 month, and 3 months after cataract surgery was performed by using OCTA. We measured the macular thickness (MT), superficial capillary plexus (SCP), deep capillary plexuses (DCP), and foveal avascular zone (FAZ) in the 3 × 3 mm macular images.

**Results:**

A total of 32 eyes of 32 type 2 diabetic patients and 40 eyes of 40 nondiabetic patients were assessed. There was a significant increase in MT at 1 month and 3 months after surgery in both groups (all *P* < 0.05), but there was no significant difference between the two groups (*P*=0.217). At 3 months postoperatively, the SCP level in the diabetic group was significantly higher than that at baseline (*P* < 0.05). The MT and SCP were negatively correlated with logMAR best-corrected visual acuity (BCVA), while the FAZ area and perimeter were positively correlated with logMAR BCVA in the diabetic group.

**Conclusions:**

Our study demonstrated that phacoemulsification can increase macular thickness in both diabetic and nondiabetic patients and increase SCP in diabetic patients within 3 months after surgery. Whether these changes persist for a longer period still needs to be investigated.

## 1. Introduction

Diabetes mellitus (DM) is one of the most prevalent diseases, affecting 425 million people worldwide [1]. Diabetes causes many ocular complications, such as cataracts and diabetic retinopathy, which are the main causes of decreased vision in diabetic patients and often coexist [2]. Cataracts occur more frequently and earlier in diabetic patients than in nondiabetic patients. Phacoemulsification surgery can not only improve visual acuity but also help doctors detect diabetic retinopathy in the early stage [3]. However, some studies have shown that cataract surgery can accelerate the progression of diabetic retinopathy (DR), and the incidence of macular edema is significantly higher in diabetic patients [4, 5]. Studies have reported the use of anti-VEGF therapy to prevent postoperative macular edema in diabetic patients with good results [6–8]. However, the reasons for the risk of DR and diabetic macular edema (DME) progression after cataract surgery are still unclear.

Optical coherence tomography (OCT angiography, OCTA) is a new and noninvasive vascular imaging technique that was developed in recent years. A special algorithm is used to perform a continuous OCT scan and obtain blood flow signals, which leads to better visualization and quantification of the retinal vessels in different layers and nonperfused areas of the macula and nerve [9, 10]. OCTA has been used to detect microvascular changes early in diabetic patients, even before clinical signs appeared [11]. In this study, we used OCTA to assess the macular thickness, superficial and deep vascular densities, foveal avascular zone (FAZ) area, perimeter, and acircularity index (AI) in diabetic and nondiabetic patients after cataract surgery. The aims of our study were to compare the OCTA parameters obtained from patients with or without diabetes after uncomplicated cataract surgery and to analyze the effects of phacoemulsification surgery on DR.

## 2. Methods

### 2.1. Patients

This prospective study enrolled 72 cataract patients with or without diabetes who planned to undergo routine phacoemulsification surgery with intraocular lens (IOL) implantation in the Ophthalmology Department of Shanghai Tenth People's Hospital between October 2017 and March 2018. This study was approved by the Institutional Review Board and followed the tenets of the Declaration of Helsinki. All patients provided written informed consent.

The inclusion criteria were patients who underwent cataract surgery with or without type 2 diabetes mellitus, an intraocular pressure (IOP) between 10 mm and 21 mm Hg, and an axial length (AL) between 20.0 mm and 26.0 mm. The exclusion criteria were as follows: (1) patients with proliferative diabetic retinopathy (PDR) or clinically significant diabetic macular edema (CSDME); (2) patients treated with intravitreal anti-VEGF drugs or panretinal photocoagulation (PRP); (3) patients with a history of ocular trauma, intraocular surgery, glaucoma, and uveitis; and (4) patients with an OCT image scan with a quality score of <5 due to severe cataracts or unstable fixation.

### 2.2. Examinations

All patients underwent complete ophthalmologic examinations, including the measurement of best-corrected visual acuity (BCVA) in logarithms of the minimum angle of resolution (logMAR), slit-lamp examination, intraocular pressure measurement, dilated fundus examination, and macular OCTA measurement at baseline (up to two weeks before surgery). Cataract severity was assessed using the lens opacities classification system III (LOCS scale). We also recorded the systolic blood pressure (SBP), diastolic blood pressure (DBP), total cholesterol (TC) level, hemoglobin A1c (HbA1c) level, and duration of diabetes.

### 2.3. Procedures

All patients underwent phacoemulsification surgery with intraocular lens (IOL) implantation. All cataract surgeries were performed using an Infiniti® vision system (Alcon Laboratories, Inc., USA) by one surgeon. The aspheric posterior chamber IOL (Tecnis ZCB00, Abbott Medical Optics, Inc., USA) was used for all surgeries. The bottle height and cumulative dissipated energy (CDE) data in each surgery were recorded. The bottle height was set at 75 cm or 90 cm, according to the stability of the anterior chamber during surgery and the patient's subjective comfort. All patients in our study received levofloxacin and pranoprofen eye drops after cataract surgery for 2 weeks and there was no difference between the two groups. The patients were followed up and underwent ophthalmic examination including BCVA, slit lamp, IOP, and OCTA at one day, one week, one month, and three months after surgery.

### 2.4. OCTA Data Collection

All microvasculature parameters were measured using OCTA at baseline and one day, one week, one month, and three months after cataract surgery. The OCTA images were obtained using a spectral-domain OCT device (RTVue-XR Avanti, version 2017.1, OptoVue, Inc., USA). Macular OCTA images of 3 × 3 mm were obtained. Macular thickness (MT) was assessed by the same OCT system at the same time as was the retinal vasculature. The full retinal thickness was measured from the inner limiting membrane (ILM) to the retinal pigment epithelium (RPE). The vessel densities of the SCP and DCP were measured, and the DCP values were calculated after the projection artifacts were removed from the SCP. The foveal avascular zone (FAZ) measurements included the FAZ area (in mm^2^), perimeter (in mm), and acircularity. The acircularity index (AI) is defined as the ratio of the perimeter of the FAZ to the perimeter of a circle with an equal area. All parameters were measured automatically.

### 2.5. Statistical Analysis

All the data were analyzed using SPSS 17.0 for Windows statistical software (SPSS, Inc., Chicago, IL, USA) and are expressed as the mean ± standard deviation (SD). The Mann–Whitney *U* test was used to evaluate the differences between the two groups. The preoperative and postoperative measurements were compared using the repeated measures analysis of variance tests with Bonferroni corrections. Pearson's correlation analyses were performed to determine the relationships between the magnitude of change in MT, SCP, and DCP from baseline and related factors, including the duration of DM, HbA1C, SBP, DBP, TC, and CDE and bottle height during cataract surgery. The correlations between BCVA and MT, SCP, DCP, and FAZ were analyzed by Spearman's correlation. *P* < 0.05 was considered statistically significant [12].

## 3. Results

In total, 32 eyes of 32 type 2 diabetic patients and 40 eyes of 40 nondiabetic patients were included in this study. In the diabetic group, there were 3 mild NPDR cases, 1 moderate NPDR case, 1 severe NPDR case, and 27 background DR cases. None of the patients had CSDME. The baseline characteristics of the patients in both groups are shown in Table 1. There were no significant differences in any of the demographic or baseline characteristics between the two groups.

**Figure 1:**
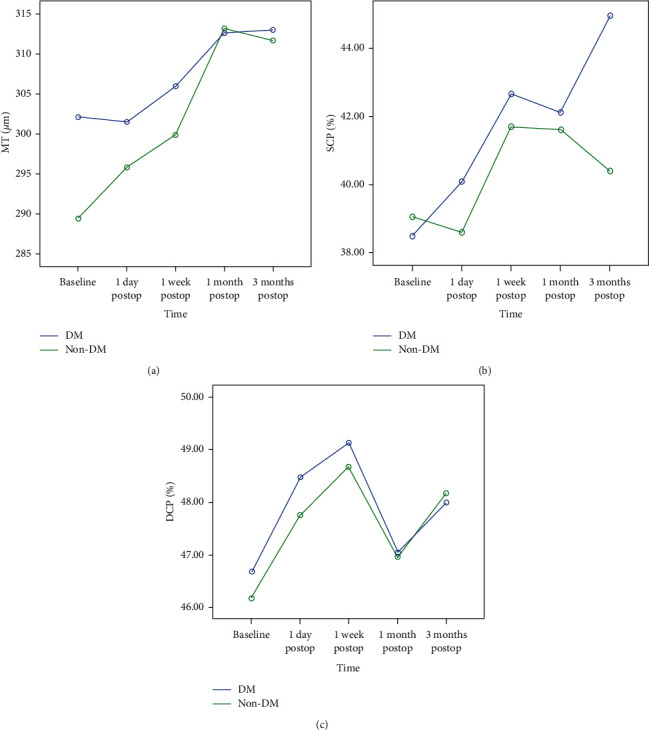
The changes in MT, SCP, and DCP after cataract surgery.

All microvasculature parameters obtained using OCTA at baseline and one day, one week, one month, and three months after cataract surgery are presented in Table 2. The changes in MT, SCP, and DCP are also shown in Figure 1. The mean MT changed from 302.09 ± 23.07 *μ*m and 289.33 ± 23.06 *μ*m at baseline to 312.64 ± 24.98 *μ*m and 313.27 ± 30.98 *μ*m at 1 month and 313.00 ± 28.16 *μ*m and 311.67 ± 28.16 *μ*m at 3 months in the diabetic and nondiabetic groups, respectively (all *P* < 0.05) (Figure 1(a)). There was no significant difference between the two groups (*P*=0.217). The SCP increased significantly from 38.47 ± 4.37% at baseline to 44.96 ± 4.52% at 3 months after surgery only in the diabetic group (*P* < 0.05) (Figure 1(b)). There were no significant differences at any postoperative timepoint or between the two groups in DCP (Figure 1(c)).

**Figure 2:**
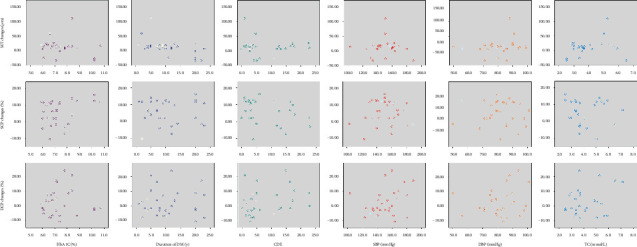
Pearson's correlation analyses between the magnitude of the changes in MT, SCP, and DCP from baseline and related factors. All *P* > 0.05. DM, diabetes mellitus; HbA1c, hemoglobin A1c; BCVA, best-corrected visual acuity; MT, macular thickness; SCP, superficial capillary plexus; DCP, deep capillary plexus; CDE, cumulative dissipated energy; SBP, systolic blood pressure; DBP, diastolic blood pressure; TC, total cholesterol.

The magnitude of change in MT, SCP, and DCP from baseline to 3 months after the surgery did not have a significant correlation with factors such as the duration of DM, HbA1C, SBP, DBP, TC, and CDE during cataract surgery (all *P* > 0.05, based on Pearson's correlation analyses) (Figure 2). The magnitude of change in MT, SCP, and DCP also did not significantly differ between bottle heights of 75 cm and 90 cm (all *P* > 0.05, based on the Mann–Whitney *U* test).

Spearman's correlation coefficients (rho) with BCVA and MT, SCP, FAZ, and PERIM in the DM group were −0.382 (*P*=0.045), −0.476 (*P*=0.012), 0.426 (*P*=0.027), and 0.382 (*P*=0.045), respectively, as shown in Table 3. The MT and SCP were negatively correlated with logMAR BCVA, while the FAZ area and perimeter were positively correlated with logMAR BCVA in the diabetic group.

**Figure 3:**
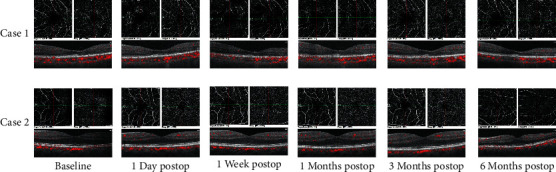
SCP, DCP, and MT images of 2 cases in the diabetic group at baseline, 1 day, 1 week, 1 month, 3 months, and 6 months after cataract surgery.

Some of our patients have completed the follow-up for 6 months after surgery.  Figure 3 shows the macular OCTA images of 2 cases in the diabetic group at baseline, 1 day, 1 week, 1 month, 3 months, and 6 months after cataract surgery. It can be seen intuitively that the macular thickness of the two diabetic patients increased at 1 and 3 months and subsided at 6 months after surgery.

## 4. Discussion

At present, cataracts and diabetes very commonly coexist. The conclusions of studies on the development of DR and DME after cataract surgery are controversial. Some studies have shown that phacoemulsification can accelerate diabetic retinopathy [4, 5, 13]. Other studies suggest that the development of DR and DME after cataract surgery is part of the natural history of the disease and that cataract surgery does not cause the progression of diabetic retinopathy [14, 15]. Although this question remains unanswered, it has been indicated in many studies that macular thickness increases after cataract surgery [16–18]. It is not yet clear whether the magnitude of change differs between diabetic and nondiabetic patients.

In our study, we used OCTA to quantitatively investigate the differences in MT, SCP, and DCP between diabetic and nondiabetic patients in the short term after phacoemulsification. Our results indicate that OCTA can effectively show retinal microvascular changes after cataract surgery in diabetic patients. We found a significant increase in MT from baseline to 1 month and 3 months postoperatively in both groups. However, there was no significant difference in the magnitude of change between the two groups. Therefore, in our short-term observation, both diabetic and nondiabetic patients exhibited an increase in MT that gradually stabilized at 3 months after surgery. It can be considered that the surgery itself did not increase the risk of DME. Cong et al. [19] summarized the possible mechanisms of macular thickness changes after cataract surgery, and the mechanisms included the release of local inflammatory mediators caused by surgical stimulation, tissue damage caused by ultrasound energy and radiation effects, surgical perfusion fluid damage, and light exposure. Although the loss of pericytes, endothelial cells, and hemodynamic abnormalities in diabetic patients can also cause postoperative macular thickness increase and even macular edema, this study did not find the difference between diabetic and nondiabetic groups from statistical analysis. It can be seen from Figure 3 that the macular thickening of these two diabetic patients at 1 and 3 months postoperatively significantly subsided at 6 months postoperatively, which further confirmed that the increase in the postoperative macular thickness was only from the operation itself, not due to the progress of diabetes. This is similar to the results reported in previous studies in nondiabetic patients. Kurt and Kilic [20] showed a decrease in MT on the first postoperative day, increases at week 1 and months 1 and 3, and a relative decrease at month 6, although MT did not return to preoperative levels. Zhao et al. [21] also reported that at 1 month and 3 months after surgery, the full retina of the fovea, parafovea, and perifovea increased significantly, and this change was more obvious in the inner layer.

Microvascular density analysis in our study showed that the SCP was significantly higher at 3 months after surgery than at baseline only in the diabetic group. No significant changes were found in DCP. This is an interesting result and may be related to pathological retinal microvascular changes in diabetic patients. Yu et al. [22] reported a significant increase in perfusion and vessel densities in both the SCP and the DCP after cataract surgery in 3 × 3 mm images. The authors considered that inflammation may impact the assessment of density parameters. Pilotto et al. [23] found that macular intermediate retinal capillary (ICP) and DCP perfusion increased at 1 day after uncomplicated cataract surgery, whereas the extent of macular SCP perfusion did not change, and all parameters almost reached baseline levels after 90 days, which seems to confirm their inflammatory nature. In our results, the diabetes group began to show a significant increase in SCP at 3 months after surgery. We believe that the changes in SCP in diabetic patients are the result of a postoperative inflammatory response, which may be due to the loss of pericytes and the increased permeability of endothelial cells in diabetic patients, ultimately leading to an increase in postoperative inflammatory factors. If this change persists for a long time, it is likely to become an important factor in the progression of DR after phacoemulsification. Therefore, from this perspective, OCTA can have a predictive effect on the progression of postoperative DR. In addition, we think there is another factor that leads to an increase in SCP. Under surgical stimulation, abnormalities in retinal hemodynamics in diabetic patients cause microcirculation disturbances and increase the compensation of the superficial vasculature, which may be beneficial to diabetic patients in the short term, and long-term follow-ups are required. Moreover, we found that DCP increased at 1 day and 1 week after surgery and decreased at 1 month after surgery. However, the magnitude of the change was small and not statistically significant. It may be because there is a choroid-rich blood supply in the deep retina, so this surgery has little effect on DCP. In addition, most of the diabetic patients had a background or only mild retinopathy in our study, and the severity of deep blood vessel damage was not severe.

Previous studies have generally suggested that macular thickness is significantly increased in the short term after phacoemulsification, but there have been a few studies on the changes in postoperative SCP and DCP. There are also very few studies on the differences in MT, SCP, and DCP between diabetic and nondiabetic patients. Giansanti et al. [24] measured MT by OCT preoperatively and at 1, 6, 15, 30, 60, 90, and 360 days after surgery and found that MT increased significantly from day 30 after surgery in diabetic patients, reaching its maximum thickness at day 60, but it was measured only on day 360 in healthy subjects. Haleem et al. [25] showed that cystoid macular edema after phacoemulsification was equally present in both diabetic and nondiabetic patients without any retinopathy. There have been no reports on postoperative SCP and DCP in diabetic patients.

We also tried to explore factors related to postoperative MT, SCP, and DCP changes. The magnitude of the changes in MT, SCP, and DCP from baseline did not have a significant correlation with factors such as the duration of DM, HbA1C, SBP, DBP, TC, or CDE during cataract surgery. Denier et al. [26] also found that there is no linear correlation between HbA1c and central retinal thickness after cataract surgery in diabetic patients. Regarding the TC, SBP, and DBP data, due to the individual differences in the patients and the limited sample size, we did not find any correlations with the patient's OCTA changes. In our study, complicated cataract surgery was not assessed, and the bottle height did not differ considerably between the groups to ensure the stability of the anterior chamber. Therefore, CDE and bottle height have little impact on retinal microvasculature changes. To ensure high OCTA image scan and data quality, we did not include cataract patients with severe opacity, which may not be enough to have an impact. Therefore, we need to conduct more research to overcome these limitations and explore the factors that affect the thickness of the macula.

Our preliminary findings indicated that the MT and SCP were negatively correlated with logMAR BCVA, while the FAZ area and perimeter were positively correlated with logMAR BCVA in the diabetic group. These results provide information that can guide future research. The shortcoming of this study is that the patient sample size was small, and most diabetic patients had a background or only mild retinopathy. Retinal function was still in the compensatory period, so the difference with respect to nondiabetic patients was not obvious. We will continue to assess more patients at different stages of DR to obtain more comprehensive results.

## 5. Conclusion

Our preliminary findings show that macular thickness increased in both diabetic and nondiabetic patients at 1 month and 3 months after cataract surgery, and the SCP increased only in diabetic patients at 3 months after cataract surgery. OCTA found that SCP increased in diabetic patients 3 months after cataract surgery, which may have a predictive effect on the progression of postoperative DR. Whether these changes persist for a longer period still needs to be investigated.

## Figures and Tables

**Table 1:** Baseline characteristics of the two groups.

	Diabetic group (*n* = 32)	Nondiabetic group (*n* = 40)	*P*
Age, y	68.31 ± 9.42	71.60 ± 6.85	0.103
Male patients, *n* (%)	31.25%	32.5%	0.318
Right eyes, *n* (%)	50%	52.5%	0.566
Duration of DM, y	10.38 ± 6.67	—	—
HbA1C, %	7.39 ± 1.24	—	—
BCVA, LogMAR	0.35 ± 0.22	0.27 ± 0.14	0.059
MT, *μ*m	302.09 ± 23.07	289.33 ± 23.06	0.998
SCP, %	38.47 ± 4.37	39.05 ± 6.45	0.077
DCP, %	46.68 ± 5.42	46.17 ± 3.74	0.972
FAZ, mm^2^	0.287 ± 0.120	0.389 ± 0.120	0.180
PERIM, mm	2.263 ± 0.527	2.254 ± 0.477	0.224
AI	1.20 ± 0.11	1.17 ± 0.08	0.258

Data in the table are presented as means ± standard deviation. DM, diabetes mellitus; HbA1c, hemoglobin A1c; BCVA, best-corrected visual acuity; MT, macular thickness; SCP, superficial capillary plexus; DCP, deep capillary plexus; FAZ, fovea avascular zone; PERIM, FAZ perimeter in mm; AI, acircularity index: ratio between the measured perimeter and the perimeter of the same size circular area based on the Mann–Whitney *U* test.

**Table 2:** Comparison of pre- and postoperative microvasculature parameters.

Group	Parameter	Baseline	1 day postop	1 week postop	1 month postop	3 months postop
DM	MT, *μ*m	302.09 ± 23.07	301.45 ± 22.37	306.00 ± 20.70	312.64 ± 24.98*∗*	313.00 ± 28.16*∗*
	SCP, %	38.47 ± 4.37	40.09 ± 5.59	42.67 ± 3.22	42.11 ± 3.40	44.96 ± 4.52*∗*
	DCP, %	46.68 ± 5.42	48.48 ± 3.81	49.13 ± 3.57	47.04 ± 3.50	48.00 ± 2.37
	FAZ, mm^2^	0.287 ± 0.120	0.253 ± 0.094	0.282 ± 0.153	0.290 ± 0.129	0.289 ± 0.146
	PERIM, mm	2.263 ± 0.527	2.060 ± 0.535	2.145 ± 0.580	2.220 ± 0.468	2.242 ± 0.565
	AI	1.20 ± 0.11	1.16 ± 0.10	1.17 ± 0.05	1.18 ± 0.11	1.15 ± 0.06

Non-DM	MT, *μ*m	289.33 ± 23.06	295.80 ± 20.21	299.87 ± 22.66	313.27 ± 30.98*∗*	311.67 ± 28.16*∗*
	SCP, %	39.05 ± 6.45	38.59 ± 6.59	41.70 ± 7.33	41.61 ± 7.07	40.39 ± 6.36
	DCP, %	46.17 ± 3.74	47.75 ± 4.02	48.68 ± 4.37	46.96 ± 2.87	48.10 ± 2.75
	FAZ, mm^2^	0.389 ± 0.120	0.343 ± 0.128	0.374 ± 0.142	0.363 ± 0.136	0.366 ± 0.124
	PERIM, mm	2.254 ± 0.477	2.377 ± 0.463	2.448 ± 0.514	2.430 ± 0.543	2.443 ± 0.480
	AI	1.17 ± 0.08	1.17 ± 0.06	1.14 ± 0.04	1.15 ± 0.09	1.15 ± 0.06

Data in the table are presented as means ± standard deviation.*∗P* < 0.05, based on repeated measures analysis of variance tests with Bonferroni corrections.

**Table 3:** The result of Spearman's correlation analysis.

Group			MT	SCP	DCP	FAZ	PERIM	AI
DM	BCVA	Rho	−0.382*∗*	−0.476*∗*	0.224	0.426*∗*	0.382*∗*	−0.023
		*P*	0.045*∗*	0.012*∗*	0.242	0.027*∗*	0.045*∗*	0.904
Non-DM	BCVA	Rho	0.028	−0.118	−0.250	−0.018	−0.054	0.031
		*P*	0.884	0.541	0.19	0.926	0.782	0.193

*∗P* < 0.05, based on Spearman's correlation analysis.

## Data Availability

The data used to support the findings of this study are available from the corresponding author upon request.

## References

[1] International Diabetes Federation (2017). *IDF Diabetes Atlas*.

[2] Kelkar A., Kelkar J., Mehta H., Amoaku W. (2018). Cataract surgery in diabetes mellitus: a systematic review. *Indian Journal of Ophthalmology*.

[3] Ostri C., Lund-Andersen H., Sander B., La Cour M. (2011). Phacoemulsification cataract surgery in a large cohort of diabetes patients: visual acuity outcomes and prognostic factors. *Journal of Cataract and Refractive Surgery*.

[4] Jeng C., Hsieh Y., Yang C. (2018). Development of diabetic retinopathy after cataract surgery. *PLoS One*.

[5] Baker C. W., Almukhtar T. (2013). Macular edema after cataract surgery in eyes without preoperative central-involved diabetic macular edema. *JAMA Ophthalmology*.

[6] Udaondo P., Garcia-Pous M., Garcia-Delpech S., Salom D., Diaz-Llopis M. (2011). Prophylaxis of macular edema with intravitreal ranibizumab in patients with diabetic retinopathy after cataract surgery: a pilot study. *Journal of Ophthalmology*.

[7] Chae J. B., Joe S. G., Yang S. J. (2014). Effect of combined cataract surgery and ranibizumab injection in postoperative macular edema in nonproliferative diabetic retinopathy. *Retina*.

[8] Khodabandeh A., Fadaifard S., Abdollahi A. (2018). Role of combined phacoemulsification and intravitreal injection of bevacizumab in prevention of postoperative macular edema in non-proliferative diabetic retinopathy. *Journal of Current Ophthalmology*.

[9] Kashani A. H., Chen C.-L., Gahm J. K. (2017). Optical coherence tomography angiography: a comprehensive review of current methods and clinical applications. *Progress in Retinal and Eye Research*.

[10] Lee J., Rosen R. (2016). Optical coherence tomography angiography in diabetes. *Current Diabetes Reports*.

[11] Khadamy J., Abri Aghdam K., Falavarjani K. (2018). An update on optical coherence tomography angiography in diabetic retinopathy. *Journal of Ophthalmic and Vision Research*.

[12] Le F., Azhati G., Li T. (2019). Retinal microvascular alterations after phacoemulsification in patients with diabetes evaluated using optical coherence tomography angiography. *National Library of Medicine*.

[13] Hong T., Mitchell P., de Loryn T., Rochtchina E., Cugati S., Wang J. J. (2009). Development and progression of diabetic retinopathy 12 months after phacoemulsification cataract surgery. *Ophthalmology*.

[14] Chéour M., Mazlout H., Falfoul Y. (2013). Évolution de la rétinopathie diabétique après chirurgie de la cataracte par phacoémulsification. *Journal Français d’Ophtalmologie*.

[15] Liao S. B., Ku W. C. (2003). Progression of diabetic retinopathy after phacoemulsification in diabetic patients: a three-year analysis. *Chang Gung Medical Journal*.

[16] Stunf Pukl S., Vidovic Valentincic N., Urbancic M. (2017). Visual acuity, retinal sensitivity, and macular thickness changes in diabetic patients without diabetic retinopathy after cataract surgery. *Journal of Diabetes Research*.

[17] Abdellatif M. K., Ebeid W. M. (2018). Variations in choroidal and macular thickness maps after uneventful phacoemulsification. *Seminars in Ophthalmology*.

[18] Moreira Neto C. A., Moreira Júnior C. A., Moreira A. T. R. (2015). Optical coherence tomography in patients undergoing cataract surgery. *Arquivos Brasileiros de Oftalmologia*.

[19] Cong W., Zhang Z., Hao X. (2017). Mechanism of the macular fovea thickness change after cataract surgery in diabetic patients. *International Eye Science*.

[20] Kurt A., Kilic R. (2018). The effects of uncomplicated cataract surgery on retinal layer thickness. *Journal of Ophthalmology*.

[21] Zhao Z., Wen W., Jiang C., Lu Y. (2018). Changes in macular vasculature after uncomplicated phacoemulsification surgery: optical coherence tomography angiography study. *Journal of Cataract and Refractive Surgery*.

[22] Yu S., Frueh B. E., Steinmair D. (2018). Cataract significantly influences quantitative measurements on swept-source optical coherence tomography angiography imaging. *PLoS One*.

[23] Pilotto E., Leonardi F., Stefanon G. (2019). Early retinal and choroidal OCT and OCT angiography signs of inflammation after uncomplicated cataract surgery. *British Journal of Ophthalmology*.

[24] Giansanti F., Bitossi A., Giacomelli G. (2013). Evaluation of macular thickness after uncomplicated cataract surgery using optical coherence tomography. *European Journal of Ophthalmology*.

[25] Haleem A., Saleem A., Memon S. (2017). Cystoid macular oedema after phacoemulsification with and without type 2 diabetes mellitus; a hospital-based clinical prospective trial in Karachi. *Journal of Pakistan Medical Association*.

[26] Denier C., Fajnkuchen F., Giocanti-Aurégan A. (2018). Central retinal thickness assessment in a real life setting after cataract surgery in diabetic patients. *Journal Français d’Ophtalmologie*.

